# A Kinase Anchoring Protein 9 Is a Novel Myosin VI Binding Partner That Links Myosin VI with the PKA Pathway in Myogenic Cells

**DOI:** 10.1155/2015/816019

**Published:** 2015-04-16

**Authors:** Justyna Karolczak, Magdalena Sobczak, Krzysztof Skowronek, Maria Jolanta Rędowicz

**Affiliations:** ^1^Department of Biochemistry, Nencki Institute of Experimental Biology, Polish Academy of Sciences, 3 Pasteur Street, 02-093 Warsaw, Poland; ^2^International Institute of Molecular and Cell Biology, 4 Trojden Street, 02-109 Warsaw, Poland

## Abstract

Myosin VI (MVI) is a unique motor protein moving towards the minus end of actin filaments unlike other known myosins. Its important role has recently been postulated for striated muscle and myogenic cells. Since MVI functions through interactions of C-terminal globular tail (GT) domain with tissue specific partners, we performed a search for MVI partners in myoblasts and myotubes using affinity chromatography with GST-tagged MVI-GT domain as a bait. A kinase anchoring protein 9 (AKAP9), a regulator of PKA activity, was identified by means of mass spectrometry as a possible MVI interacting partner both in undifferentiated and differentiating myoblasts and in myotubes. Coimmunoprecipitation and proximity ligation assay confirmed that both proteins could interact. MVI and AKAP9 colocalized at Rab5 containing early endosomes. Similarly to MVI, the amount of AKAP9 decreased during myoblast differentiation. However, in MVI-depleted cells, both cAMP and PKA levels were increased and a change in the MVI motor-dependent AKAP9 distribution was observed. Moreover, we found that PKA phosphorylated MVI-GT domain, thus implying functional relevance of MVI-AKAP9 interaction. We postulate that this novel interaction linking MVI with the PKA pathway could be important for targeting AKAP9-PKA complex within cells and/or providing PKA to phosphorylate MVI tail domain.

## 1. Introduction

Myosin VI (MVI) is a unique unconventional actin-based motor that unlike other known myosins moves towards the minus end (i.e., backwards) of actin filaments [1, 2]. MVI belongs to a large myosin superfamily and has a domain organization similar to other known myosins; that is, it contains a motor, neck, and tail domain [3]. Its ~140 kDa heavy chain is composed of the N-terminal motor domain (with the actin and ATP binding sites), a neck, to which two calmodulin molecules are bound, and a tail domain [1, 2, 4]. MVI exists as a monomer or a dimer, and it is believed that several factors such as cargo binding, monomer availability, and/or phosphorylation within the tail domain determine MVI heavy chain dimerization, which occurs by a helical region within the tail [5, 6]. MVI functions in numerous cellular processes through its interaction with actin (via its N-terminal motor domain) and tissue specific partner proteins (via its C-terminal globular domain, also termed a cargo domain). Two regions of the MVI cargo domain were found to be involved in partner recognition: a positively charged RRL region and a hydrophobic WWY region. Also, a positively charged cluster located within the cargo tail was shown to bind to PIP_2_-containing liposomes, possibly aiding in partner binding [2, 4]. Several tissue specific MVI binding partners have been already identified in mammals; among them are proteins engaged in the regulation of cytoskeleton dynamics, proteins associated with the Golgi apparatus and the endoplasmic reticulum, and proteins involved in endocytosis and cell adhesion as well as proteins with enzymatic activities [2, 4].

All the known mutations within* MYO6* cause sensorineural deafness [7]. Defects were also observed in the brain [8, 9], intestines [10], and kidney [11]. One of the mutations, a H246R mutation within the human MVI motor domain, was also found to be associated with hypertrophic cardiomyopathy [12] suggesting important role(s) of this motor in striated muscle. Indeed, our recent work has shown that in striated muscle as well as in myogenic cells MVI could be involved in the organization/maintenance of the sarcoplasmic reticulum, Golgi apparatus, adhesive structures (and intercalated discs in case of cardiac muscle), nuclei, and the neuromuscular junction [13–15]. We found that in skeletal muscles MVI might interact with TOM1 (target of myb1 homolog isoform 1, a protein involved in intracellular transport and autophagy), FMRP (fragile X mental retardation autosomal homolog 1, a protein involved in mRNA transport), and hnRNP proteins (involved in the RNA transport and maturation) [13].

To further understand the role of MVI in myogenic cells, we performed a search for its interaction partners in myoblasts and myotubes. It resulted in identification of AKAP9 as a novel MVI interaction partner. This ~230 kDa coiled-coil protein (also termed as yotiao) is highly expressed in cardiac and skeletal muscle, placenta, pancreas, and the brain [16]. It belongs to A kinase anchoring proteins (AKAPs) that create a compartmentalized environment inside the cell to bring various signaling molecules to their targets [17]. For example, in the heart, AKAP9 was shown to form a complex with a slowly activating potassium channel (*I*_Ks_), important for cardiac repolarization and with other enzymes, which are responsible for channel regulation such as PKA, phosphatase, adenylyl cyclase, and phosphodiesterase [18]. AKAP9 mutations cause long-QT syndrome manifested by cardiac arrhythmia [18, 19]. AKAP9 was also shown to link NMDA with the inositol 1,4,5-trisphosphate receptor type 1 [18]. There are also reports that AKAP9 could play an important role in cytoskeleton dynamics and organization by targeting the PKA kinase to the cytoskeleton, thus affecting cell motility, proliferation, and adhesion [20]. Several data indicate that AKAP9 is involved in striated muscle and synapse functioning, in development of breast and thyroid cancers, and in spermatogenesis [18, 21].

In the presented work we characterize a novel MVI-AKAP9 interaction and postulate that it could be important for linking MVI with the PKA signaling.

## 2. Materials and Methods

### 2.1. Cell Culture

C2C12 mouse myoblasts (American Type Culture Collection, USA), kindly provided by Prof. Krzysztof Zablocki from the Nencki Institute, were maintained in DMEM containing 4.5 g/L glucose and supplemented with 10% heat-inactivated fetal bovine serum (FBS), antibiotics (1000 IU/mL penicillin and 1000 UI/mL), and 4 mM L-glutamine at 37°C in humidified air containing 5% CO_2_. Differentiation was initiated upon reaching confluence (considered as day 0) by transferring to medium containing 2% horse serum (HS) instead of 10% FBS, and the culture was continued for up to the next 7–10 days. To observe acetylcholine-rich clusters, cells were differentiating in 8-well Permanox chamber slides (Sigma-Aldrich, USA) coated with laminin (Invitrogen, USA) as described by [22].

### 2.2. siRNA Knockdown of MVI

A  MVI-KD stable cell line was generated based on the pSilencer 2.1-U6 hygro vector system (Ambion Inc., USA) essentially as described by [23]. 5′-AACTACGCGATACAATCAATA-3′ siRNA sequence against a coding region of mouse MVI mRNA was used with the corresponding scrambled sequence as a negative control (5′-ATAACATACCGTACGAATAAC-3′). Notably, the chosen shRNA targeted a different MVI region than the ones used for the protein depletion in PC12 cells but evoked similar changes in the cell motility, Golgi organization, and cytoskeleton organization [23]. C2C12 cells were transfected using Lipofectamine 2000 and then selected on hygromycin B (Sigma-Aldrich, USA). MVI expression level was assessed by Western blot as well as RT-PCR.

### 2.3. C2C12 Cell Transfection

C2C12 myoblasts cultured on coverslips were transfected using Lipofectamine 2000 (Life Technologies, USA) with plasmids encoding GFP-fused human full length MVI (GFP-MVI) and its globular tail domain (GFP-MVI-GT), kindly provided by Dr. Tama Hasson from University of California, Los Angeles [24]. Also, the cells were transfected with plasmids encoding markers of vesicular organelles fused with GFP, kindly provided by Dr. S. Havrylov (McGill University, Canada). The following proteins were overexpressed: Rab5-GFP (found on clathrin-coated vesicles and early endosomes), Rab7-GFP (present on late endosomes), Rab11-GFP (present on early and recycling endosomes as well as the Golgi apparatus), and LAMP1-GFP (present on lysosomes). After 36 h of transfection, cells were fixed and stained with anti-AKAP9 and/or anti-MVI antibodies as described in Section 2.5.

### 2.4. Immunoblotting

Cells were lysed in an ice-cold buffer containing 50 mM Tris-HCl pH 7.5, 150 mM NaCl, 0,1% Triton X-100, 1 mM DTT, 1 mM EGTA, 1 mM PMSF, and complete protease inhibitor cocktail (Roche). Cell lysates (10–20 *μ*g of protein per well) were separated using 10% or 12% polyacrylamide SDS-gels and then transferred to a nitrocellulose membrane. Protein concentration was determined by Bio-Rad protein assay reagent (Bio-Rad, USA). MVI was detected with a rabbit polyclonal antibody raised against porcine MVI heavy chain (Proteus, USA) at 1 : 500 dilution. AKAP9 and DOCK7 were detected with goat polyclonal anti-AKAP9 (Abcam, UK) and anti-DOCK7 (Santa Cruz, USA) antibodies, both at 1 : 100 dilution. PKA and phospho-PKA (pPKA) were detected with mouse anti-PKA and rabbit anti-pPKA antibodies, respectively (both from Santa Cruz), at 1 : 500 dilutions. Mouse monoclonal antibody against *β*-actin (from Sigma-Aldrich, USA) at 1 : 1000 dilution was used to detect *β*-actin. *γ*-actin and GAPDH (glyceraldehyde 3-phosphodehydrogenase) were detected with sheep polyclonal anti-*γ*-actin and mouse monoclonal anti-GAPDH antibodies (both from Merck Millipore, USA), at 1 : 500 and 1 : 10,000 dilutions, respectively. Anti-mouse, anti-sheep, and anti-rabbit antibodies conjugated with horse radish peroxidase (all diluted at 1 : 10,000) were applied for detection of primary antibodies using the ECL system (Pierce, USA). Western blot was performed and followed by densitometric analysis. Student's *t*-test was used to evaluate the quantitative data.

### 2.5. Immunolocalization Studies

Cells on cover slips were fixed in 4% formaldehyde in phosphate-buffered saline pH 7.4 (PBS) for 20 min at room temperature, washed with PBS, blocked in 2% horse serum, and permeabilized with 0.02% Triton X-100 in PBS for 30 min at room temperature. Cover slips were then incubated for 2 h at room temperature or overnight at 4°C with anti-MVI (Proteus, USA) antibody diluted at 1 : 50 and anti-AKAP9 antibody (Abcam, UK) diluted at 1 : 200 and washed with PBS, followed by cell incubation with 1 *μ*g/mL Alexa Flour 488-conjugated goat anti-rabbit IgG or Alexa Fluor 555-conjugated donkey anti-goat IgG (both from Molecular Probes, Invitrogen, USA). Vectashield mounting medium (Vector Labs, USA) was used to mount the slides. Alexa488-conjugated bungarotoxin (BTX, from Invitrogen, USA) was used to stain acetylcholine-rich clusters. For negative controls, the primary antibodies were omitted. Images were collected as described in [13]. Unless stated otherwise, the images represent the confocal 0.3-*μ*m sections of the cell center. MetaMorph software (Leica MM AF Basis Offline Version 1.4.0) was used for the quantification studies.

### 2.6. Proximity Ligation Assay (PLA)

The assay was performed according to the manufacturer's instructions (Olink Bioscience, Sweden). Briefly, myoblasts and mature myotubes after fixation were blocked in Duolink blocking solution in a humidity chamber for 30 min at 37°C and incubated with primary antibodies: polyclonal anti-MVI (1 : 50) and anti-AKAP (1 : 200) in Duolink antibody diluent solution for 3 h at 37°C. Cells were next washed twice in a wash buffer for 5 min at room temperature. For secondary antibodies conjugated with oligonucleotides, PLA probe anti-goat MINUS and PLA probe anti-rabbit PLUS were applied in Duolink antibody diluent solution for 1 h at 37°C and washed twice with a wash buffer for 5 min. Duolink assay was further performed strictly according to the manufacturer's instructions. For negative controls, the primary antibodies were omitted. Also, a control assay was performed on the scrambled and MVI-KD cells, which revealed about 30% reduction of the positive signals in the knockdown cells that notably express several times more AKAP9 than the scrambled cells (not shown).

### 2.7. Coimmunoprecipitation

To perform coimmunoprecipitation, HEK 293 cells transiently transfected with pEGFP-C3-MVI FL+S+ construct (encoding full length human MVI, a gift from Dr. Tama Hasson from University of California, Los Angeles) were lysed in a buffer containing 50 mM Tris (pH 7.5), 150 mM NaCl, 2 mM EDTA, 0.2% Triton X-100, 2 mM MgCl_2_, 2 mM MgATP, 50 mM NaF, and 1 mM Na_3_VO_4_ and supplemented with the complete protease inhibitor cocktail. The lysates were precleared with A/G magnetic beads (Pierce, USA) for 2 h at 4°C and subsequently incubated with 10 *μ*g of the anti-MVI antibody or normal rabbit serum as a control overnight at 4°C. The beads were extensively washed with lysis buffer and then subjected to SDS-PAGE. Western blot was performed using anti-AKAP9 (1 : 200) or anti-DOCK7 (1 : 200) antibodies to detect the coimmunoprecipitated MVI-AKAP9 or MVI-DOCK7 complexes.

### 2.8. GST Pull-Down

A fusion protein composed of GST and the MVI C-terminal globular tail domain (GST-MVI-GT) as well as GST alone was purified as described in [13]. Cells were lysed at three time points during differentiation (days 0, 3, and 7) in ice-cold buffer containing 50 mM Tris (pH 7.5), 150 mM NaCl, 0.5% Triton X-100, 1 mM DTT, 5 mM EGTA, 50 mM NaF, 1 mM Na_3_VO_4_, and 0.5 mM PMSF and supplemented with the complete protease inhibitor cocktail. Samples were precleared with GST-bound Glutathione Sepharose 4B beads for 2 hours at 4°C to remove proteins nonspecifically binding to Glutathione Sepharose 4B or to GST and subsequently incubated with Glutathione Sepharose 4B beads bound with GST-MVI fusion protein or GST alone. The beads were exhaustively washed in the ice-cold buffer described above. Myoblast (and myotube) proteins associated with GST-MVI-GT or with GST were subjected to SDS-PAGE electrophoresis.

### 2.9. Sample Preparation and Protein Identification by LC-MS/MS

After the electrophoretic separation of samples obtained in the pull-down assay, the gel was stained with Coomassie R-250 and equal pieces were cut from the experimental (GST-fused MVI globular tail, GST-MVI-GT) and control (GST alone) gel lanes. Proteins from gel slices were subjected to the standard procedure of in-gel digestion with trypsin (Thermo, USA) and extraction according to manufacturer recommendations. Eluted peptides were loaded on an EASY-nLC II nano-LC system equipped with Acclaim PepMap100 Nano Trap Column 2 cm × 200 *μ*m C-18 precolumn and EASY Column 10 cm × 75 *μ*m C-18. The solvent system consisted of 0.1% formic acid in water (solvent A) and 0.1% formic acid in acetonitrile (solvent B). Samples were chromatographed using a flow rate of 300 nl/min with a two-step linear gradient of 1–40% B from 0–20 min, 40–100% B from 20–30 min, and 100% B from 30–35 min. The chromatographic eluent was directly conducted into the amaZone ion trap mass spectrometer (Bruker) working in the regime of data-dependent MS to MS/MS switch. The raw data preprocessing with Data Analysis software (version 2.1.1, Bruker) resulted in peak lists which were used to search the Swiss Prot protein database using the Mascot search engine (version 2.3.01, Matrix Science, London, UK) with the following search parameters: taxonomy restriction: Mus musculus (mouse), enzyme specificity: trypsin, permitted number of missed cleavages: 2, fixed modification: carbamidomethylation (C), variable modifications: phosphorylation (S,T,Y) and oxidation (M), protein mass: unrestricted, peptide mass tolerance: ±0.6 Da, and fragment mass tolerance: ±0.6 Da. Only the proteins that met our criteria (i.e., (i) present only in the GST-MVI-GT sample but not in the control sample, (ii) identified by at least three distinct peptide spectra, and (iii) with Mascot score ≥50) were considered the potential MVI-binding partners. MVI peptides detected in the sample were excluded from the analysis.

### 2.10. Estimation of cAMP Levels

For estimation of cAMP levels, the untreated scrambled and MVI-KD cells were cultured for 48 h and then washed twice with PBS, dissolved in sample diluent of total cAMP enzyme immunoassay kit (Biotrend, Germany), and the further procedure followed the supplier protocol. The experiment was performed twice in duplicate.

### 2.11. PKA Phosphorylation of the MVI Globular Tail Domain

Exogenous catalytic subunit of PKA (from Sigma-Aldrich, USA) was used to phosphorylate the GST-MVI-GT domain. After dialysis against 50 mM Tris, pH 7.4, 50 mM KCl, 5 mM MgCl_2_, and 1 mM DTT, GST-MVI-GT (1 *μ*g) and GST (1 *μ*g) were then incubated for 1 h at 30°C with the kinase domain (2 units per sample), which prior to the experiment was equilibrated as recommended by the supplier in the presence of 5 mM ^32^P-*γ*ATP (10 mCi/mL, Hartmann Analytic GmbH, Germany). The reaction was stopped with the addition of 4x SDS sample buffer (0.25 mM Tris-HCl, pH 6.8, 5% *β*-mercaptoethanol, 5% SDS, 40% glycerol, and 0.08% bromophenol blue) followed by incubation at 100°C for 3 min. The samples were then subjected to 10% polyacrylamide SDS gel electrophoresis followed by autoradiography.

### 2.12. Statistical Analysis

Data are presented as mean ± SD; all *P* values were calculated by two-sided Student's *t*-test. The difference was considered to be statistically significant at the level of *P* < 0.05.

## 3. Results

### 3.1. AKAP9 Was Identified as a Potential MVI Binding Partner

We have previously shown that MVI plays important roles both in skeletal and cardiac muscle [13, 15] and in C2C12 myoblasts [14]. To further explore MVI function in myogenic cells, we performed a search for its binding partners by means of an affinity chromatography with the GST-tagged globular tail domain of MVI (GST-MVI-GT) used as a bait. The eluates were subjected to tandem mass spectrometry. The analysis was performed in undifferentiated (day 0) and differentiating (day 3 and day 7) myoblasts. Samples from the GST-bound resin served as the control. It should be emphasized that with this method one cannot discriminate whether the identified proteins bind directly or indirectly to the MVI cargo domain.

**Figure 1:**
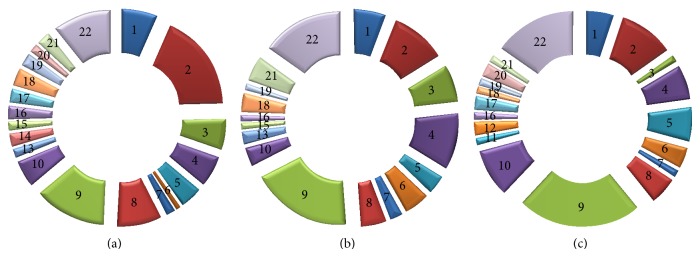
Potential binding partners of MVI identified by pull-down assay and mass spectrometry. (a) Protein groups identified in day 0 myoblasts, (b) proteins identified in the intermediate stage (day 3 myoblasts), and (c) proteins identified in day 7 myotubes. (1) Replication and DNA repair. (2) Transcription. (3) RNA processing. (4) Protein biosynthesis. (5) Protein modifications and transport. (6) Protein degradation. (7) Ion transport. (8) Cytoskeleton. (9) Signal transduction. (10) Metabolism. (11) Cellular respiration. (12) Peroxisome. (13) Endocytosis. (14) Exocytosis. (15) Stress. (16) Autophagy. (17) Apoptosis. (18) Adhesion. (19) Cell junctions. (20) Cell movement/migration. (21) Cell division. (22) Unknown function.

We obtained a total list of ~250 potential novel MVI interaction partners. There were proteins found only in proliferating myoblasts (77), only in 3-day myoblasts (55), and only in myotubes (51). Only nine proteins were found in all three examined samples, among them were A kinase anchoring protein (AKAP9) and talin (the constituent of adhesive complexes). Interaction of MVI and talin is to be described elsewhere (Karolczak et al., unpublished). The identified proteins for each examined sample were grouped based on their known functions depicted by the Uniprot database (Figure 1). For all three samples, the most abundant were proteins involved in transcription (30 proteins), in signal transduction (42 proteins), and in the organization of the cytoskeleton (16 proteins). However, all groups varied both quantitatively and qualitatively depending on the differentiation stage. The number of the detected proteins involved in the numerous cellular functions in three differentiation stages has been presented in Table 1.

As it was mentioned above, AKAP9, a regulator of the PKA kinase activity, was found in all three examined samples. In day 0 myoblasts, three AKAP9 peptides were detected, seven in day 3 myoblasts, and four in the myotubes.

### 3.2. AKAP9 Is Expressed in Undifferentiated and Differentiating Myoblasts

**Figure 2:**
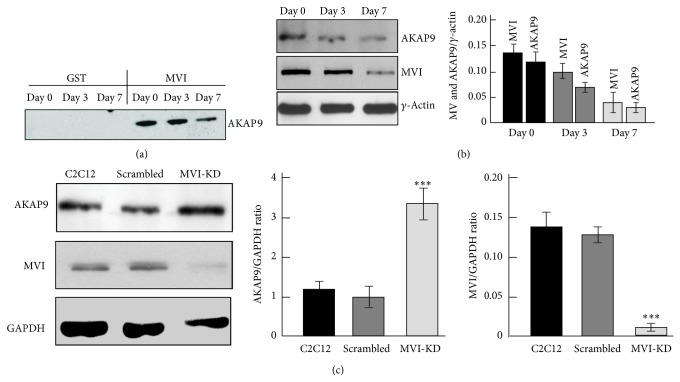
AKAP9 expression in myogenic cells probed by immunodetection. (a) AKAP9 was found in the pull-downs of the day 0, day 3, and day 7 myoblast samples. (b) AKAP9 expression is decreasing during myoblast differentiation into myotubes. Right panel: quantitative analysis of the AKAP9 and MVI content with respect to the level of *γ*-actin. (c) The level of AKAP9 expression is substantially elevated in MVI-KD cells when compared with untreated C2C12 and scrambled myoblasts. Right panels: quantitative analyses of the AKAP9 and MVI content with respect to the level of glyceraldehyde-3-phosphate dehydrogenase (GAPDH). The results in (b) and (c) are presented as means ± SD from two experiments; ^∗∗∗^ *P* < 0.001.

The presence of AKAP9 in all three samples was confirmed by Western blotting with an antibody against the C-terminal region of the protein. As shown in Figure 2(a), the protein was detected in samples from the GST-GT-MVI resin but not from the GST resin. Interestingly, the amount of AKAP9 decreased during myoblast differentiation (similarly to MVI) as revealed by the immunoblot analysis (Figure 2(b)). We also checked that the AKAP9 level in MVI-KD myoblasts with MVI expression decreased down to <10%. Western blotting followed by densitometric analysis revealed that the level of AKAP9 was increased severalfold in MVI-KD cells (Figure 2(c)).

### 3.3. AKAP9 and MVI Colocalize in Myoblasts and Myotubes

**Figure 3:**
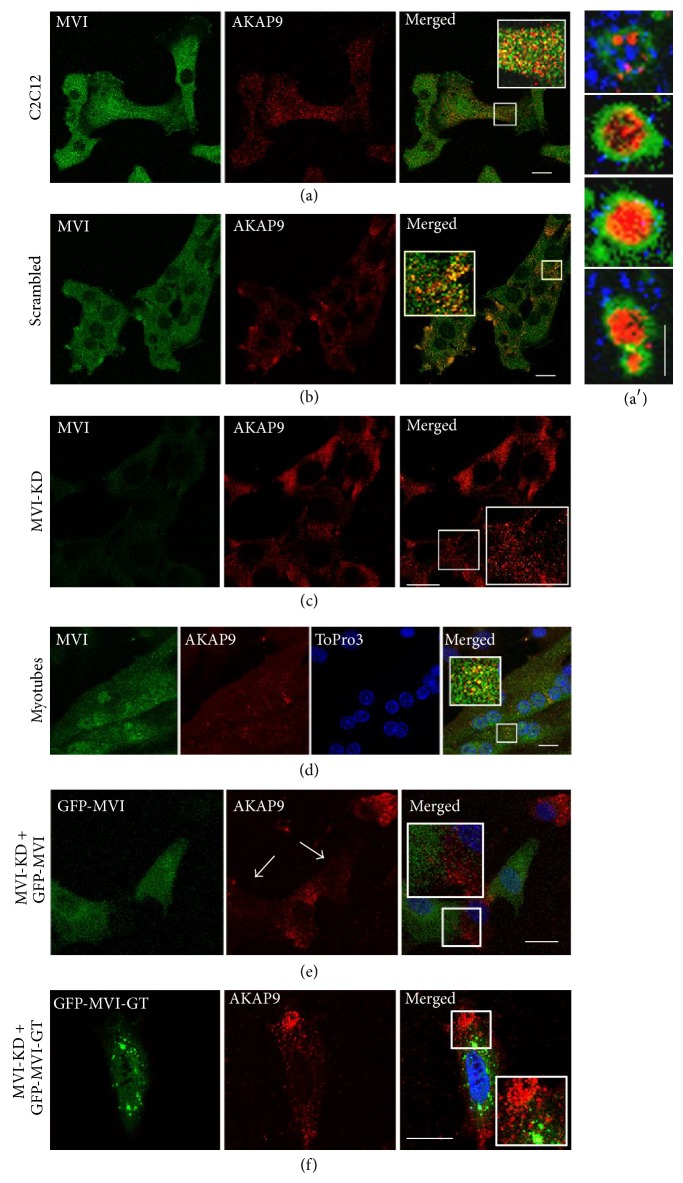
AKAP9 and MVI colocalization in undifferentiated myoblasts and in myotubes by means of confocal microscopy. (a) Untreated C2C12 myoblasts (C2C12), (b) scrambled cells, (c) MVI-depleted cells (MVI-KD), and (d) day 7 myotubes. (e) MVI-KD cells expressing GFP-MVI and (f) MVI-KD cells expressing GFP-tagged MVI globular tail domain (GFP-MVI-GT). MVI in (a)–(d) was visualized with anti-MVI antibody (in green) and in (e)-(f) by the GFP fluorescence and AKAP9 with anti-AKAP9 antibody (in red) and nuclei (in (d)) with ToPro3 (in blue). Insets, 2-3x magnification of the areas marked in the merged panels. (a′) AKAP9 (stained with anti-AKAP9 antibody, in red) and MVI (stained with anti-MVI antibody in blue) were associated with early endosomes containing overexpressed Rab5-GFP (in green). Images of the central cell section (*z* = 0.3*μ*m) were obtained with a Leica confocal microscope. Bars: in (a)–(f), 20*μ*m and in (a′), 2*μ*m.

MVI and AKAP9 colocalized in undifferentiated control and scrambled myoblasts in numerous puncti scattered throughout the cytoplasm (Figures 3(a) and 3(b), insets). To identify the MVI and AKAP9 containing puncti, we overexpressed GFP-fused constructs encoding proteins markers of endocytic and lysosomal compartments such as Rab5, Rab7, Rab11, and LAMP11. Out of them, AKAP9 localized only to Rab5-GFP containing vesicles that were also associated with MVI-stained puncti (Figure 3(a′)) and not with the other vesicle markers (not shown). These data indicate that MVI and AKAP9 colocalization is on clathrin-coated pits and early endosomes [25].

In MVI-KD cells, the protein was also visible throughout the cytoplasm with increased signal at the cell edges (Figure 3(c)). Noticeably, AKAP9-associated fluorescence intensity was evidently higher in MVI-KD cells, confirming the Western blot analysis (see Figure 2(c)) and indicating that MVI knockdown upregulated its expression.

MVI and AKAP9 colocalization was also visible in mature myotubes (Figure 3(d)). AKAP9-associated staining was evidently weaker due to its lower amount in the myotubes (see Figure 2(b)) but most of AKAP9 puncti were associated with MVI (Figure 3(d), inset).

To confirm that enhanced AKAP9 synthesis was indeed associated with less MVI, we performed a rescue experiment, in which the MVI-KD cells were transfected with a plasmid encoding GFP-fused full length MVI (GFP-MVI). In the cells expressing GFP-MVI (marked with arrows), AKAP9-associated staining was substantially lower in comparison with nontransfected MVI-KD cells (Figure 3(e), inset).

To check whether AKAP9 localization depends on MVI motor activity, we transfected MVI-KD myoblasts with a plasmid encoding the MVI globular tail domain fused with GFP (GFP-MVI-GT). As shown in Figure 3(f), unlike expression of the full length MVI, expression of the tail domain did not affect AKAP9 localization. Also, this MVI domain lacking motor activity was present in the perinuclear region in large aggregate-like puncti not associated with AKAP9 staining (Figure 3(f), inset).

### 3.4. Assessment of an AKAP9-MVI Interaction

Since the data presented above indicate that MVI and AKAP9 may interact with each other, we decided to further assess this association by means of coimmunoprecipitation as well as by the proximity ligation assay (PLA), which allows for* in situ* detection of two proteins that exist within close intracellular proximity (within the 20–40 nm range). The positive PLA signal is considered an evidence for interaction of two given proteins (and/or their domains) [26].

**Figure 4:**
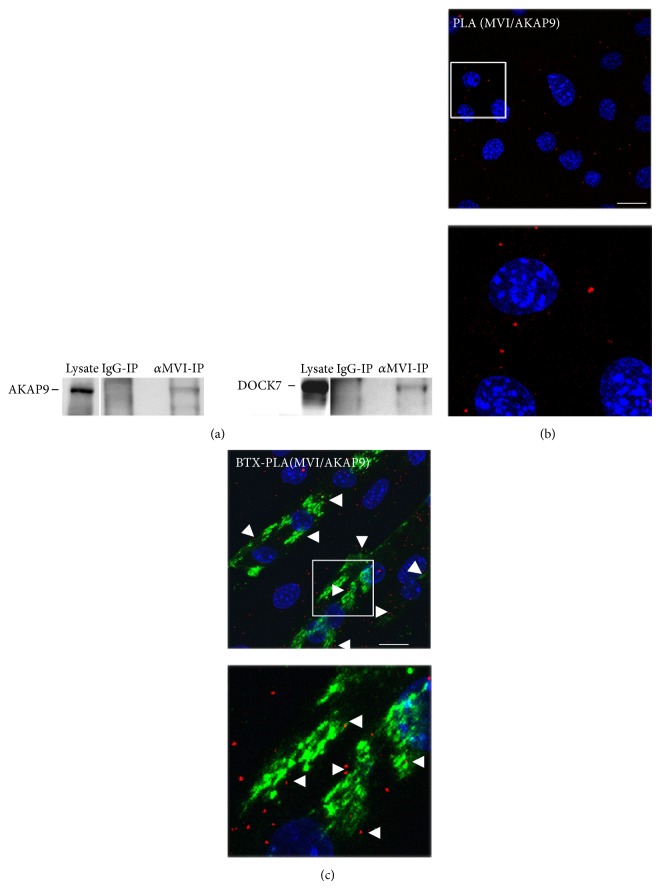
Validating the AKAP9 and MVI interaction. (a) Coimmunoprecipitation of AKAP9 (left panel) and DOCK7 (right panel) with anti-MVI antibody from HEK 293 cells overexpressing GFP-tagged full length human MVI heavy chain. Left lanes: cell lysate (lysate), middle lanes: samples precipitated with a normal rabbit serum (IgG-IP), and right lanes: *α*MVI-IP. Samples precipitated with anti-MVI antibody were probed with either anti-AKAP9 or anti-DOCK7 antibodies as marked on the figure. PLA assay probing MVI and AKAP9 interactions (in red) in undifferentiated myoblasts (b) and in myotubes (c). In blue, nuclei stained with DAPI. (c) MVI-AKAP9 possible interactions are also seen close to bungarotoxin- (BTX-) stained acetylcholine-rich clusters (in green) marked by arrowheads. The lower panels in (b) and (c) are ~3x magnifications of the areas marked in (b) and (c). Images in (b) and (c) of the cell central sections (*z* = 0.3*μ*m) were obtained with a Leica confocal microscope. Bars: 20*μ*m.

Coimmunoprecipitation with the anti-MVI antibody revealed the presence of AKAP9 in the precipitate from lysates of HEK293 cells overexpressing GFP-tagged MVI construct, thus confirming the presence of both proteins in the precipitate (Figure 4(a), left panel). As a positive control, the precipitate was also probed with anti-DOCK7 antibody (Figure 4(a), right panel) as we previously showed DOCK7-MVI interaction in PC12 cells [27].

MVI-AKAP9 interaction was further visualized* in situ *by the PLA assay which revealed the presence of numerous puncti (in red) resembling the interaction sites (Figures 4(b) and 4(c)). Both proteins were in very close proximity not only in undifferentiated myoblasts (Figure 4(b)) but also in mature myotubes (Figure 4(c)). Moreover, in myotubes, this interaction was detected in the vicinity of nascent acetylcholine-rich clusters as well (Figure 4(c), arrowheads).

The control PLA assay performed on the scrambled and MVI-KD myoblasts revealed that significantly more positive PLA signals were detected in the scrambled cells than in the knockdown cells (~60% reduction) despite the fact that in MVI-KD cells the amount of AKAP9 was substantially elevated (not shown).

### 3.5. Depletion of MVI Affects cAMP Level

**Figure 5:**
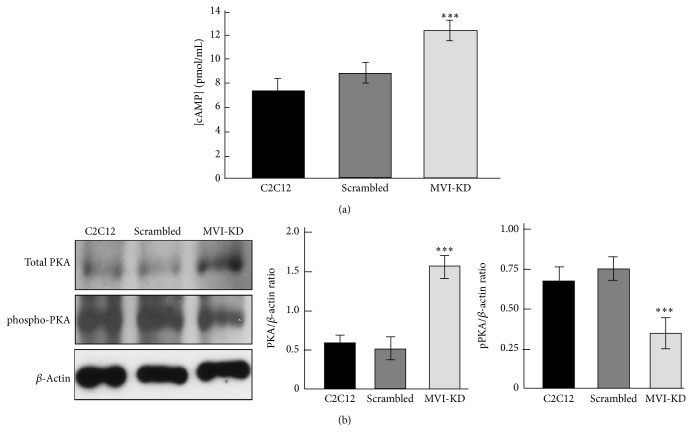
MVI depletion affects cAMP and PKA levels in C2C12 myoblasts. (a) The amount of cAMP (presented as pmol/mL) in untreated C2C12, scrambled and MVI-KD myoblasts was estimated as described in Section 2. (b) Probing the levels of total (upper panel) and phosphorylated (lower panel) PKA in untreated C2C12, scrambled and MVI-KD myoblasts by means of Western blot technique with the respective antibodies. Right panels: quantitative analysis of the PKA and phospho-PKA content with respect to the level of*β*-actin. The results in (a) and (b) are presented as a mean ± SD from two experiments (for (a) run in duplicate); ^∗∗∗^ *P* < 0.001.

A substantial increase of AKAP9 expression and a change in its distribution in MVI-KD myoblasts imply that there could be a link between the AKAP9-MVI complex and PKA kinase activity which is dependent on the intracellular level of cyclic AMP (cAMP). It is noteworthy that a decrease in the level of cAMP is required for initiation of myoblast differentiation into myotubes and acetylcholinesterase expression [28]. In order to understand the mechanism of the MVI-AKAP9 interaction, we checked whether MVI depletion could affect the intracellular level of cAMP. With the use of a total cAMP enzyme immunoassay, we found that in MVI-KD myoblasts the level of cAMP was substantially elevated with respect to both untreated (increase by about 40%) and scrambled (increase by about 30%) cells, suggesting a MVI-dependent change in cAMP metabolism. This increase was accompanied by a threefold increase of PKA expression as assessed with an anti-PKA antibody, detecting the total amount of kinase regardless of its phosphorylated state (Figure 5(b)). However, when the same samples were probed with an anti-phospho-PKA antibody detecting (auto)phosphorylated kinase, the intensity of the band corresponding to phosphorylated kinase was ~50% decreased with respect to the untreated and scrambled cells (Figure 5(b)). Thus MVI depletion is associated with an increase in PKA level; however, a smaller kinase fraction is phosphorylated.

### 3.6. MVI Globular Tail Is a Substrate for PKA

**Figure 6:**
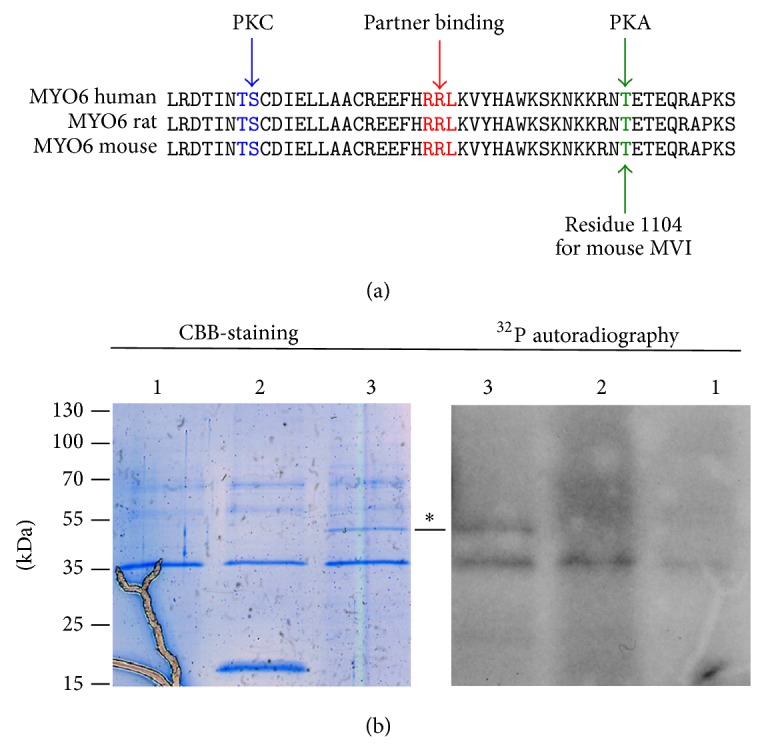
Myosin VI is a target of PKA. (a) A putative PKA phosphorylation site (threonine residue 1104 in the mouse MVI heavy chain, in green) is located in the vicinity of a very conserved region within the RRL partner recognition cluster (in red). In blue, the putative PKC phosphorylation sites. The kinase specific phosphorylation sites were depicted with a NetPhosK 1.0 Server (http://www.cbs.dtu.dk/services/NetPhosK/). The MVI sequences used for analyses are human (Q9UM54), rat (D4A5I9), and mouse (Q64331). (b)* In vitro* PKA phosphorylation assay. Left panel: Coomassie brilliant blue stained gel (CBB-staining), right panel: ^32^P autoradiogram. Lane 1: the catalytic kinase domain; lane 2: GST; lane 3: GST-tagged MVI globular tail domain. ∗ Points to the ~52-kDa band of interest (GST-MVI domain).

MVI harbors a putative PKA phosphorylation site at threonine residue 1104 for mouse, 1100 for rat, and 1134 for human myosin as depicted with the NetPhos bioinformatic tool [29]. This residue is located in a very conserved region of the globular tail domain of numerous MVI heavy chains (Figure 6(a)) raising a possibility that MVI could be a substrate for PKA. Thus the interaction described above could be important not only for targeting the AKAP9-PKA complex but also for providing the kinase for MVI phosphorylation.

We addressed this hypothesis by performing an* in vitro *phosphorylation assay using GST-tagged MVI cargo domain and commercially available PKA catalytic subunit; GST alone was used as a control (Figure 6(b)). As shown in Figure 6(b), a ~52-kDa band corresponding to the recombinant GST-MVI tail domain incorporated radioactive ^32^P resulting from the kinase assisted hydrolysis of [*γ* ^32^P]ATP. No incorporation was observed in a ~20-kDa band corresponding to GST indicating that it was the MVI but not the GST moiety that was phosphorylated by the PKA kinase subunit. The bands corresponding to the kinase catalytic subunit also incorporated radioactive ^32^P due to its autophosphorylation. Noticeably, kinase autophosphorylation was higher in the presence of GST and the MVI globular tail.

## 4. Discussion

In the study on myogenic cells presented herein, we identified AKAP9, a regulator of PKA kinase activity, as a novel MVI binding partner and showed that this interaction could be functionally relevant.

AKAP9 was found in pull-down samples of undifferentiated and differentiating myoblasts and mature myotubes but its amount—similarly to MVI—was decreasing during myoblast differentiation into myotubes. Noticeably, the protein is also present in mature skeletal and cardiac muscle where it is termed yotiao [16]. In skeletal muscle AKAP9 is predominantly localized subjacent to acetylcholine receptors in the neuromuscular junction and was seen within the Z-line [16], and in cardiac muscle it has been recently identified as a gene associated with long-QT syndrome (LQTS), manifested by cardiac arrhythmia [18]. Interestingly, in patients with the H246R mutation in MVI that presented symptoms of dilated cardiomyopathy, a prolonged QT interval was also observed in their ECG pattern [12].

The question arises of the functional relevance of the MVI-AKAP9 interaction taking into consideration that the main role of AKAP9 (and other members of the AKAP family) is to regulate PKA kinase activity and to ensure that the activated PKA acts only on substrates that reside close to the AKAPs [30]. At least two main complementary mechanisms could be proposed to understand this novel interaction. First MVI motor activity could be necessary to target the AKAP9-PKA complex to its final destination(s). The second one assumes that the MVI-AKAP9 interaction is required to bring PKA into the vicinity of MVI so the kinase could phosphorylate the MVI tail.

MVI binding partners interact with one out of two partner recognition sites (one charged and one hydrophobic) and this binding is believed to be important for cargo transport or its anchoring to the actin cytoskeleton (see [4]). We do not know yet which of the MVI binding regions is involved in the association with AKAP9, but certainly this interaction is functional because depletion of MVI causes not only an increase of AKAP9 and PKA kinase expression levels but also a substantial increase in the cAMP level. Moreover, MVI motor-dependent changes in the distribution of AKAP9 were observed, indicating the importance of MVI motor activity for AKAP9 targeting. Interestingly, a wild-type phenotype regarding the AKAP9 localization and expression was rescued by overexpression of full length MVI and not by the globular tail domain lacking the motor domain. In our opinion, impaired AKAP9 targeting resulting from MVI depletion could be compensated by an increase of the AKAP9-PKA complex expression. For example, a compensatory effect was shown in* Saccharomyces cerevisiae*, in which increased chitin synthesis compensated a “stress response” mechanism induced by abnormal cell wall assembly due to class II myosin deficiency [31].

Several potential phosphorylation sites within the MVI cargo domain as well as a number of serine/threonine kinases that might be able to phosphorylate the MVI tail (including PKC and PKA) have been depicted by the NetPhosK 1.0 bioinformatic server [29]. It has been postulated that phosphorylation of the cargo domain could affect MVI heavy chain dimerization and cargo binding but so far no biochemical data confirming this putative phosphorylation have been reported [4, 32]. The server found one putative PKA phosphorylation threonine residue (see Figure 6(a), marked in green) located within a very conserved region of the MVI tail and is also evolutionarily conserved. For the purpose of this study, the human, rat, and mouse regions were shown (Figure 6(a)). This potentially phosphorylatable threonine residue is only 14 residues apart (towards C-terminus) from the charged RRL region involved in partner recognition. Moreover, there is also a putative conserved PKC recognition site(s) on the N-terminal flank of the RRL region (Figure 6(a)). It is therefore possible that phosphorylation of either of these sites could affect a net charge value and have impact on binding of a given cargo. Thus it could be employed by a cell to regulate the cargo binding, dependent on cargo, myosin, and kinase availability. Neither PKA nor PKC have been detected in the examined eluates though there is a report in which tyrosine LMTK-2 kinase (lemur tyrosine kinase 2) binds to MVI in HeLa cells [33]. However, unlike for MVI and PKA, there are no reports on MVI phosphorylation by LMTK-2 kinase. Noticeably, a direct interaction between the myosin V (MV) tail and CaMKII kinase associated with phosphorylation of MV Ser1650 was shown to be important for cargo dissociation and MV translocation to the nucleus [34, 35].

The observation that the MVI-AKAP9 complex was also present in myotubes at acetylcholine-rich clusters further confirms our earlier suggestion that MVI (by interaction with its muscle specific partners, e.g., with AKAP9) could play important roles in neuromuscular junction development as both MVI and AKAP9 were shown to be important for brain and muscle synapse functioning [13, 16]. It is noteworthy that PKA-associated involvement of another unconventional myosin V in the neuromuscular junction has been already shown [36, 37].

## 5. Conclusion 

The newly identified interaction between MVI and AKAP9 seems to have functional relevance in myotube formation and neuromuscular junction development and may link MVI with cAMP-dependent PKA signaling, which is crucial for myoblast differentiation. The observation that the highest concentration of AKAP9 (and MVI) was shown in undifferentiated myoblasts and the lowest in mature myotubes is consistent with the data showing that the level of cAMP and the activities of its downstream signaling molecules decrease during myotube formation [28]. Also, it could explain why we experienced difficulties with myotube formation by MVI-depleted myoblasts [14], which—as we found out now—showed substantially higher cAMP levels than the control cells. Also, the question arises as of whether and how AKAP9 regulates MVI phosphorylation by PKA as well as whether and how PKA affects interaction between AKAP9 and MVI. We postulate that the novel MVI-AKAP9 interaction could also be important in cardiac muscle since, on one hand, AKAP9 was shown to play a crucial role in the proper functioning of the heart [18] and, on the other hand, mutated MVI was shown to be associated with hypertrophic cardiomyopathy [12]. Pharmacological targeting of this interaction could be considered as a potential antiarrhythmia or antihypertrophy therapy.

## Figures and Tables

**Table 1:** The number of MVI interaction partners involved in different cellular functions during myoblast differentiation.

Number	Function	Day 0	Day 3	Day 7
1	Replication and DNA repair	8	4	5
2	Transcription	22	8	9
3	RNA processing	7	1	6
4	Protein biosynthesis	7	5	9
5	Protein modifications and transport	5	5	3
6	Protein degradation	1	3	4
7	Ion transport	2	1	2
8	Cytoskeleton	10	4	4
9	Signal transduction	16	18	16
10	Metabolism	6	7	3
11	Cellular respiration	0	1	0
12	Peroxisomes	0	2	0
13	Endocytosis	2	0	2
14	Exocytosis	3	0	0
15	Stress	2	0	1
16	Autophagy	3	1	1
17	Apoptosis	3	2	0
18	Adhesion	4	1	3
19	Cell junctions	3	1	1
20	Cell movement/migration	2	2	0
21	Cell division	3	1	4
22	Unknown function	13	12	13
